# Antibody-oligonucleotide conjugates for spatial proteomics: principles, applications, and challenges: Antibody-oligonucleotide conjugates for spatial proteomics

**DOI:** 10.3724/abbs.2025212

**Published:** 2025-11-27

**Authors:** Yinghui Qiu, Chunlan Li, Peiying Ye, Haiyun Zhang, Yanxiu Liu, Weiyan Ma, Chen Lin, Rongqin Ke

**Affiliations:** 1 School of Medicine Huaqiao University Xiamen 362021 China; 2 School of Material Sciences Huaqiao University Xiamen 362021 China; 3 Department of Thoracic Surgery Xiamen Chang Gung Memorial Hospital Xiamen 361000 China

**Keywords:** spatial proteomics, antibody-oligonucleotide conjugates, tissue microenvironment, multi-omics

## Abstract

Spatial biology aims to elucidate cellular organization, function, and interactions within native tissue contexts, offering key insights into both normal physiology and disease. Spatial proteomics complements this by enabling high-resolution mapping of protein localization and abundance, directly reflecting functional cellular states. Unlike transcriptomics, which infers potential activity, proteomics captures actual molecular functions, including post-translational modifications and dynamic interactions. However, *in situ* protein profiling poses significant challenges, as proteins cannot be directly sequenced or easily targeted via nucleic acid hybridization. Antibody-oligonucleotide conjugates (AOCs) address this limitation by converting protein recognition into a DNA-based readout, thereby enabling sensitive and scalable detection. In this review, we outline the core principles of AOC-based spatial proteomic technologies, including multiplexed protein analysis, *in situ* protein-protein interactions, and integration with other biomolecular data. We highlight their applications in decoding tissue complexity and disease pathology and examine key technical challenges that remain. Overall, AOCs offer distinct advantages, including DNA-mediated signal amplification, spatially resolved proteomic profiling, and compatibility with multi-omics approaches, positioning them as powerful platforms in the advancement of spatial biology.

## Introduction

The spatial organization of proteins within eukaryotic cells governs essential biological processes, such as gene expression, signal transduction, and apoptosis [1]. Protein mislocalization is implicated in various diseases, including neurodegenerative disorders, cancer, and metabolic dysfunctions [2]. Characterizing protein distribution at the subcellular level is therefore critical for deciphering disease mechanisms and identifying potential therapeutic targets. Spatial proteomics has emerged as a powerful approach, enabling high-resolution mapping of protein distribution in intact tissues and single cells. Unlike traditional proteomics, which quantifies protein abundance, spatial proteomics reveals cellular heterogeneity by mapping subcellular localization and protein-protein interactions (PPIs), thereby enabling the precise characterization of cellular functions and disease progression [3].

Traditional methods, such as mass spectrometry (MS), immunohistochemistry (IHC), and immunofluorescence (IF), have advanced the field but still face limitations in spatial resolution, multiplexing capacity, and sensitivity [ 4– 6] . In response, several emerging modalities have been developed, including aptamer-based platforms that leverage synthetic oligonucleotide binders for protein detection [7]. While aptamers offer advantages in chemical stability and *in vitro* selection, antibody-oligonucleotide conjugates (AOCs) combine the high specificity and affinity of antibodies with the versatility of DNA-based detection, enabling highly multiplexed and spatially resolved proteomic profiling.

This review summarizes AOC-based spatial proteomics technologies, covering their design, including antibody selection, site-specific conjugation, and DNA-based amplification. Key technologies such as multiplexed protein and PPI detection are highlighted. Applications related to the cellular architecture, immune microenvironments, and disease signaling are discussed. Despite these advances, challenges persist in terms of reproducibility, conjugation efficiency, and imaging compatibility. Future directions should focus on enhancing scalability, standardization, and integration with multi-omics and computational methods to advance spatial proteomics.

## AOC Design: from Antibody Engineering to Signal Amplification

The core principle of AOCs lies in their dual-functional architecture, which integrates the high specificity of antibodies with the versatility of oligonucleotides. This hybrid structure allows AOCs to retain the antigen-binding affinity of antibodies while leveraging DNA for signal amplification, molecular encoding, and precise quantification [8]. A key advantage of AOCs is their ability to enhance detection sensitivity through signal amplification. Upon binding to the target antigen, the oligonucleotide component, typically a single-stranded DNA (ssDNA) oligonucleotide, can serve as a template for various amplification strategies, thereby dramatically increasing the sensitivity and efficacy of detection [9]. This feature enables AOCs to detect low-abundance proteins that would otherwise remain undetectable using conventional immunoassays. In addition to enhancing sensitivity, the DNA oligonucleotide serves as a molecular barcode, facilitating the unique identification and quantification of target proteins in downstream assays [10].

### Antibody selection

In AOC-based workflows, optimizing the conjugation efficiency between antibodies and DNA is crucial, as it directly impacts specificity and sensitivity in subsequent experimental steps [11]. Selecting the appropriate antibody requires careful consideration of multiple factors to ensure the functionality and overall experimental success of the conjugate. The specificity and affinity of an antibody are the primary determinants of its performance [12]. High affinity is essential for maintaining strong binding interactions, particularly under low-concentration conditions [ 13, 14] . The source and subclass of the antibody also play crucial roles. Monoclonal antibodies are generally preferred because of their high specificity and batch-to-batch consistency, which improves reproducibility in conjugation experiments. While polyclonal antibodies recognize multiple epitopes on a target, they may introduce variability [14].

Different antibody subtypes ( *e*. *g*., IgG vs IgM) affect conjugation efficiency and stability. IgG is the most commonly used subtype because of its structural stability, whereas IgM, despite its high valency, presents challenges in terms of conjugation due to its large size and complex structure [15]. Therefore, alternative antibody formats have gained attention. Nanobodies, derived from camelid heavy-chain-only antibodies, are small (~15 kDa), highly soluble, and chemically stable, allowing them to access epitopes that are inaccessible to IgG and tolerate harsh conjugation conditions [16]. Similarly, variable new antigen receptors (VNAR) from sharks are the smallest naturally occurring antibody-like molecules (~12 kDa) and are capable of targeting cryptic epitopes with remarkable stability; however, their use is limited by commercial availability [17]. In addition, recombinant fragments such as fragment antigen-binding (Fab) and single-chain fragment variable (scFv) fragments offer modularity. They can be engineered for site-specific conjugation using tags such as His-tags or sortase motifs, thereby improving orientation and reducing non-specific modifications [ 18, 19] . While these alternative formats offer advantages in terms of size, flexibility, and stability, they may exhibit a lower intrinsic affinity or require customized screening and expression pipelines ( Supplementary Table S1).

**Figure 1:**
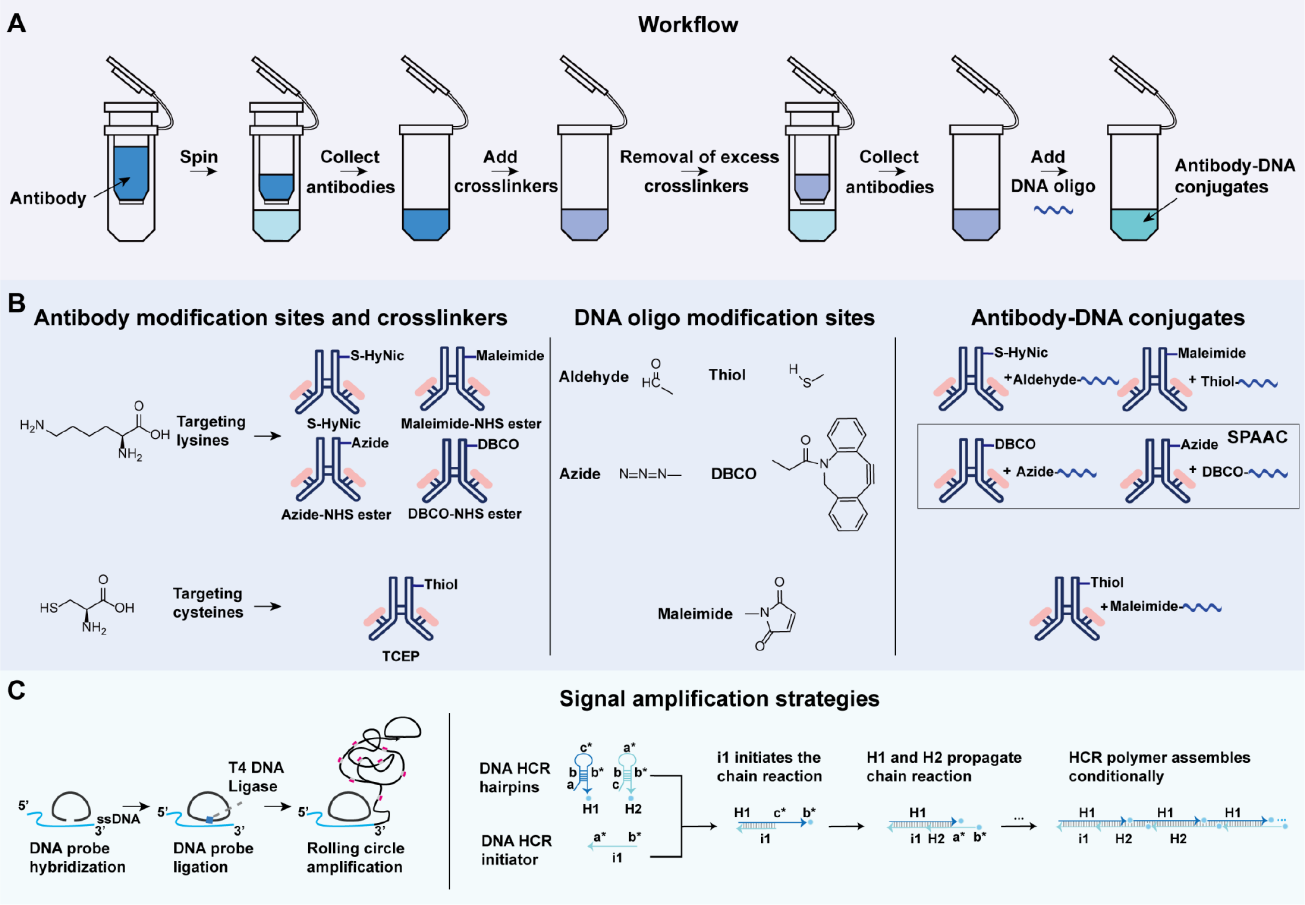
Figure 1 AOC preparation and signal amplification strategies (A) AOC synthesis workflow: antibody concentration and purification, activation of antibodies with crosslinkers, and conjugation of functionalized antibodies to oligonucleotides. (B) Overview of AOC synthesis chemistry: antibody modification sites are activated by different crosslinkers and subsequently react with correspondingly modified DNA oligonucleotides to generate AOCs, including those via SPAAC click chemistry. (C) AOC signal amplification strategy: an ssDNA hybridizes with a phosphorylated padlock probe via complementary base pairing, which is circularized by T4 DNA ligase. RCA then uses the circular DNA template and a primer with a free 3′-OH end; DNA polymerase extends the primer around the circle, producing a long ssDNA with repeated sequences for signal amplification. HCR is a one-step, isothermal, enzyme-free amplification method in which kinetically trapped hairpins (H1 and H2) remain metastable until triggered by a cognate initiator strand (i1). The initiator opens one hairpin, exposing a sequence that sequentially opens others, initiating a self-assembly cascade that forms long nicked double-stranded DNA polymers.

Antibody stability is another crucial factor, as it must remain functional throughout the conjugation process. Stability under various pH, temperature, and storage conditions should be evaluated, as repeated freeze-thaw cycles can lead to degradation and loss of activity [20]. The purity of the antibody significantly influences the conjugation efficiency. High-purity antibodies reduce interference from contaminants and enhance the success of coupling [21]. In addition, standard commercial antibody preparations often contain stabilizing additives such as sodium azide, bovine serum albumin, or glycerol, which can interfere with chemical crosslinking [22]. While removal via ultrafiltration or desalting is possible, it risks the loss of a portion of the antibodies. To avoid additional purification steps, it is advisable to purchase antibodies that are affinity-purified or stored in minimal buffer solutions [ 23, 24] . Given that each conjugation reaction leads to some degree of antibody loss, starting with 50–100 μg of purified antibody to obtain a sufficient final product is recommended [25] ( Figure 1A). Finally, supply chain sustainability should be considered to ensure a consistent and long-term antibody source, which is crucial for experimental reproducibility [26].

### Conjugation strategies for AOCs: from site-specific labelling to biorthogonal click chemistry

A critical aspect of AOCs is the efficient and stable attachment of DNA to antibodies without compromising their target-binding capabilities. Traditional random conjugation of DNA labels to antibodies often leads to structural distortions, compromising antigen-binding affinity and increasing the risk of nonspecific interactions [27]. To address these challenges, two major strategies have been developed: site-specific chemical conjugation and biorthogonal click chemistry.

Site-specific conjugation methods typically target amino acid residues such as lysine and cysteine. Lysine conjugation utilizes N-hydroxysuccinimide (NHS) ester chemistry, which reacts with lysine residues on the antibody surface to form stable amide bonds [28]. This method is simple, cost-effective, and widely used in traditional oligonucleotide labelling. However, since lysine residues are abundantly distributed on antibodies (~40 exposed sites in IgG), conjugation occurs randomly, leading to heterogeneous labelling and potential steric hindrance that may affect antigen-binding specificity [22]. To minimize these issues, PEG-based linkers are often introduced to reduce steric effects and enhance antibody stability [29].

Cysteine conjugation employs maleimide-thiol chemistry, which results in the formation of stable thioether bonds with thiol groups on cysteine residues. Reducing agents, such as dithiothreitol (DTT) or tris(2-carboxyethyl)phosphine (TCEP), are often used to maintain cysteine residues in their reduced, reactive form [ 25, 30] . However, cysteine residues are relatively scarce (~12 per IgG molecule) and are often buried within the tertiary structure of an antibody. Therefore, selective reduction is required to expose accessible thiol groups without compromising structural integrity [31]. This strategy offers improved conjugation specificity and reproducibility but requires careful optimization to avoid antibody denaturation [32]. Although lysine conjugation is simpler and achieves higher labelling efficiency, cysteine conjugation offers superior site specificity and better preserves antibody functionality ( Figure 1B and Supplementary Table S2).

Click chemistry has emerged as a powerful bioconjugation strategy because of its high specificity, biocompatibility, and efficiency under mild reaction conditions [ 33, 34] . Among the various click-based approaches, strain-promoted azide-alkyne cycloaddition (SPAAC) is the most widely adopted in AOC synthesis, enabling selective, copper-free conjugation between azide- and cyclooctyne-modified antibodies and DNA while preserving antibody functionality [35]. In addition to SPAAC, other copper-free click reactions offer unique advantages. The inverse electron demand Diels-Alder (IEDDA) reaction is particularly attractive for live-cell and *in vivo* applications, as it facilitates rapid and bioorthogonal conjugation without the need for toxic catalysts [36]. Strain-promoted alkyne-nitrone cycloaddition (SPANC) is another promising strategy that combines high reactivity and stability at low concentrations, making it particularly suitable for intracellular bioconjugation [ 37, 38] .

Emerging supramolecular and catalytic click strategies, such as cucurbituril-catalyzed azide-alkyne cycloaddition (CB-AAC) and cooperative capture synthesis (CCS), expand the toolbox of novel mechanisms. While CB-AAC enables mechanically interlocked structures, its slow kinetics limit its broader use [39]. CCS accelerates the reaction rate and broadens the substrate scope by incorporating cyclodextrin as a catalyst, forming a hydrogen-bonding network that enhances the reaction efficiency [40]. Despite their promise, these methods remain in early adoption stages. Additionally, noncovalent conjugation systems, such as the biotin-streptavidin interaction, offer modular and reversible alternatives, particularly for assay development and signal amplification [41]. Among current strategies, SPAAC remains the most established owing to its efficiency, biocompatibility, and ease of integration into AOC workflows ( Supplementary Table S3).

### Limitations of traditional signal amplification methods and the shift toward DNA-based strategies

Traditional protein detection methods, including IHC and tyramide signal amplification (TSA), remain the standard approaches for assessing protein expression in tissue sections in both research and diagnostic pathology [42]. IHC provides valuable spatial context for protein localization but relies on relatively low levels of signal amplification, which can limit its sensitivity for detecting low-abundance targets. TSA enhances signal intensity by leveraging horseradish peroxidase (HRP)-mediated enzymatic deposition of labelled tyramides at the site of antigen-antibody binding. However, despite its improved sensitivity, TSA is still constrained by its limited multiplexing capability due to spectral overlap and the need for sequential detection steps.

To overcome the limitations of signal sensitivity in AOC-based workflows, DNA-based amplification strategies have been integrated into *in situ* detection platforms. Common DNA amplification techniques include polymerase chain reaction (PCR) [43], loop-mediated isothermal amplification (LAMP) [44], rolling circle amplification (RCA) [45], and hybridization chain reaction (HCR) [46]. PCR remains the gold standard for nucleic acid amplification due to its high sensitivity and specificity. However, its requirement for repeated thermal cycling poses significant challenges for *in situ* applications. The elevated temperatures needed for DNA denaturation can disrupt tissue morphology and compromise protein conformation, making PCR unsuitable for spatial proteomic analyses that require preservation of native cellular architecture [ 43, 47] . LAMP, while offering high amplification efficiency under isothermal conditions, operates at relatively high temperatures and produces complex amplification products, which currently limits its compatibility with tissue-based spatial analyses. In contrast, RCA provides a robust, isothermal amplification method that generates long ssDNA concatemers through localized polymerase-driven extension. This technique achieves single-molecule sensitivity without compromising tissue integrity, making it highly compatible with spatial transcriptomic and proteomic applications [45]. HCR offers an enzyme-free amplification approach in which a self-assembling DNA hybridization cascade is initiated only upon target recognition. This localized, programmable reaction confines signal amplification to the vicinity of the target site, enabling nanometer-scale spatial resolution while minimizing the off-target background [48]. In summary, RCA and HCR are most compatible with AOC-based workflows, as they offer robust signal amplification while preserving tissue integrity and spatial resolution. ( Figure 1C and Supplementary Table S4).

## AOC-based Spatial Proteomics: Imaging & Multi-omics Integration

Traditional multiplexed imaging techniques are often constrained by spectral overlap, photobleaching, and limited multiplexing capacity, typically allowing the detection of no more than five proteins per cycle [49]. To overcome these limitations, advanced methods such as imaging mass cytometry (IMC) and cyclic IF (CycIF) have been developed. While IMC achieves high-resolution imaging through metal isotope-labelled antibodies, its scalability is constrained by two factors: the limited availability of isotopes and the time-consuming nature of point-by-point scanning [50]. CycIF increases multiplexing capacity through repeated cycles of staining and signal removal; however, the iterative workflow is time-consuming and poses a risk of antigen degradation over multiple rounds [51]. Click chemistry-based TSA further enhances detection sensitivity and allows for the profiling of up to 28 proteins per cycle in formalin-fixed, paraffin-embedded (FFPE) tissues [52]. Nevertheless, like CycIF, it relies on sequential fluorophore cleavage and antibody stripping, limiting its throughput and scalability.

### Multiplexed protein detection via AOCs

In contrast, AOC-based imaging leverages the programmability and flexibility of DNA barcoding to overcome many of these constraints. AOC-based spatial proteomics platforms enable highly multiplexed protein detection, PPI analysis, and simultaneous imaging of proteins with other biomolecules, offering high specificity, spatial resolution, and broad applicability in both biological and clinical research ( Supplementary Table S5).

**Figure 2:**
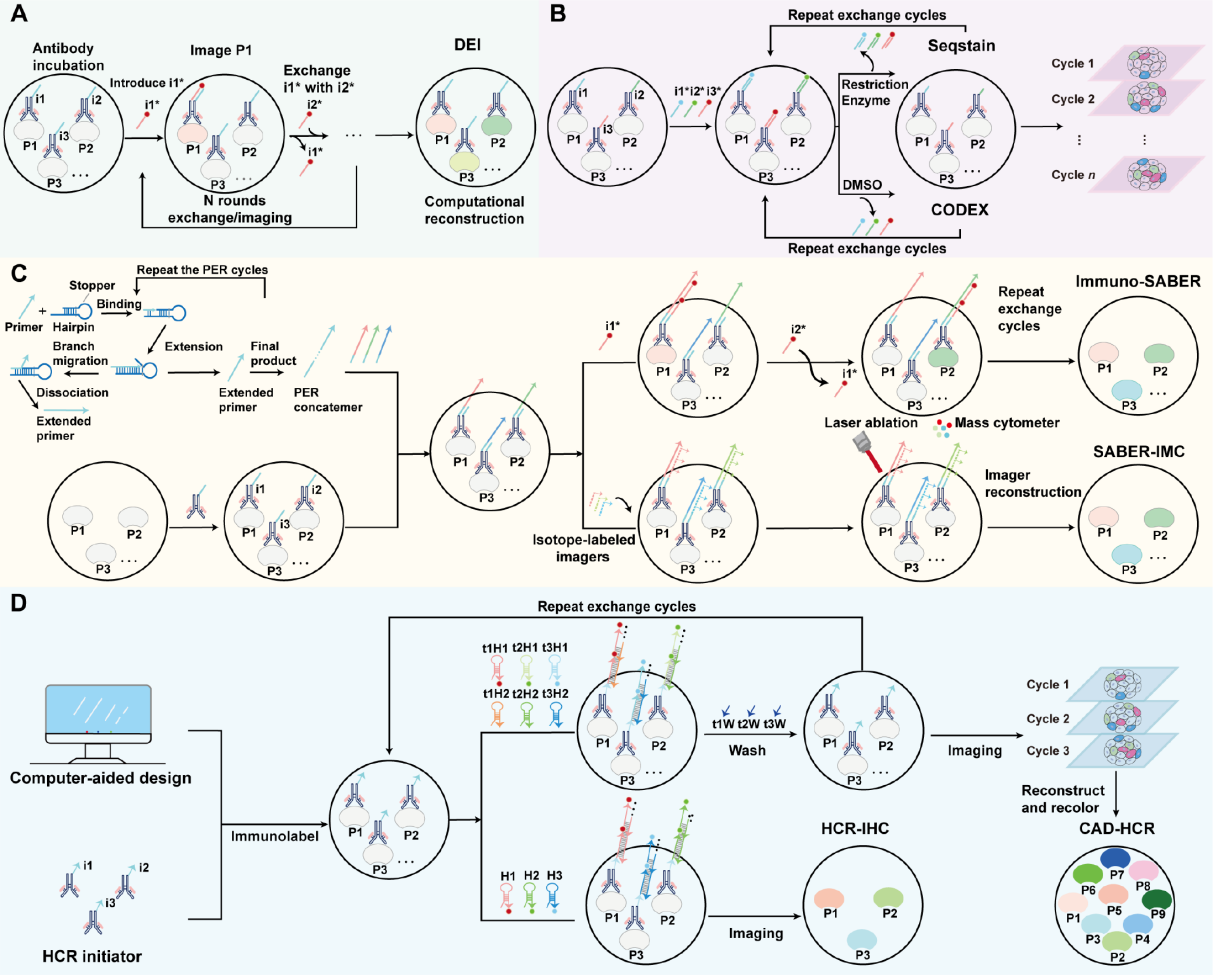
Figure 2 Principle of multiplex protein detection technology based on AOCs (A) DEI: target proteins (P1, P2, ..., Pn) are labelled in a single step via antibodies conjugated to orthogonal DNA docking strands (i1, i2, ..., in). The fluorescence imager strands (i1*, i2*, ..., in*) sequentially hybridize to visualize the targets and are rapidly washed away. Computational alignment merges images with pseudocolors assigned to generate the final composite image. (B) SeqStain and CODEX: SeqStain employs cyclic immunostaining with fluorescent DNA-labelled antibodies, followed by nuclease digestion, imaging, and computational reconstruction to generate spatial maps. CODEX enables multiplexed imaging of FFPE or fresh frozen tissues through iterative antibody staining, chemical stripping, hybridization, and re-patterning, preserving the tissue architecture. (C) Immuno-SABER and SABER-IMC: immuno-SABER uses DNA-barcoded antibodies and concatemer-based signal amplification for highly multiplexed imaging. SABER-IMC integrates this approach with metal-labelled DNA for IMC, allowing multiplexed protein detection without spectral limitations and minimizing autofluorescence and photobleaching. (D) HCR-IHC and CAD-HCR: HCR-IHC enhances IHC through HCR, where AOCs trigger the self-assembly of DNA polymers for signal amplification. CAD-HCR enhances specificity and multiplexing by utilizing computationally designed DNA sequences, facilitating precise detection, reducing crosstalk, and reversible signal removal for cyclic imaging.

SeqStain employs nucleases to cleave fluorescent signals from DNA-labelled antibodies under mild conditions, thereby preserving tissue integrity while allowing for multiple imaging rounds [53]. However, similar to CycIF, it still relies on iterative staining, which limits its efficiency for high-plex profiling. To shorten imaging time, DNA exchange imaging (DEI) [54] and co-detection by indexing (CODEX) [ 25, 55] incorporate DNA-barcoded antibodies, enabling the rapid binding and stripping of fluorescent oligonucleotides ( Figure 2A,B). By facilitating fluorescence exchange rather than requiring repeated antibody staining, these methods reduce the imaging time from weeks to just a few days. Nevertheless, both remain affected by tissue autofluorescence and the limited dynamic range in complex tissues.

Further improvements in AOC-based multiplexed imaging have been achieved by integrating DNA signal amplification strategies to increase the detection sensitivity and reduce the number of imaging cycles [56]. Immunostaining with signal amplification by exchange reaction (Immuno-SABER) utilizes short DNA primers to initiate the formation of DNA hairpin structures, which amplify fluorescence signals by generating multiple binding sites for complementary fluorescent oligonucleotides [57]. This amplification strategy increases signal intensity while minimizing the need for iterative staining, allowing for higher throughput and improved accuracy ( Figure 2C). Unlike DEI, which relies on ssDNA hybridization for fluorescence readout, Immuno-SABER employs repetitive DNA barcode sequences that yield stable, amplified signals across multiple targets [57]. Building upon this concept, SABER-IMC combines DNA-based signal amplification with metal-labelled DNA sequences for IMC, eliminating the constraints of fluorescence-based detection. SABER-IMC avoids issues such as autofluorescence and photobleaching, enabling stable, high-sensitivity imaging for high-resolution spatial proteomics, whereas Immuno-SABER can extend such imaging to relatively thick tissue samples [58] ( Figure 2C).

Beyond these advancements, computational approaches have further refined AOC-based multiplexed imaging. Computer-aided design-HCR (CAD-HCR) optimizes AOC specificity by generating an extensive library of computationally designed DNA barcodes [59]. In this system, the DNA component of the AOC serves as a molecular barcode that can be selectively amplified through HCR. Unlike conventional fluorescence-based methods, which are prone to irreversible signal degradation and photobleaching, CAD-HCR enables reversible signal amplification, allowing repeated cycles of staining and destaining without compromising sample integrity. This reversibility significantly enhances sample reusability, making it particularly valuable for cyclic imaging applications in spatial proteomics ( Figure 2D).

### AOC-based proximity approaches for *in situ* profiling of PPIs

**Figure 3:**
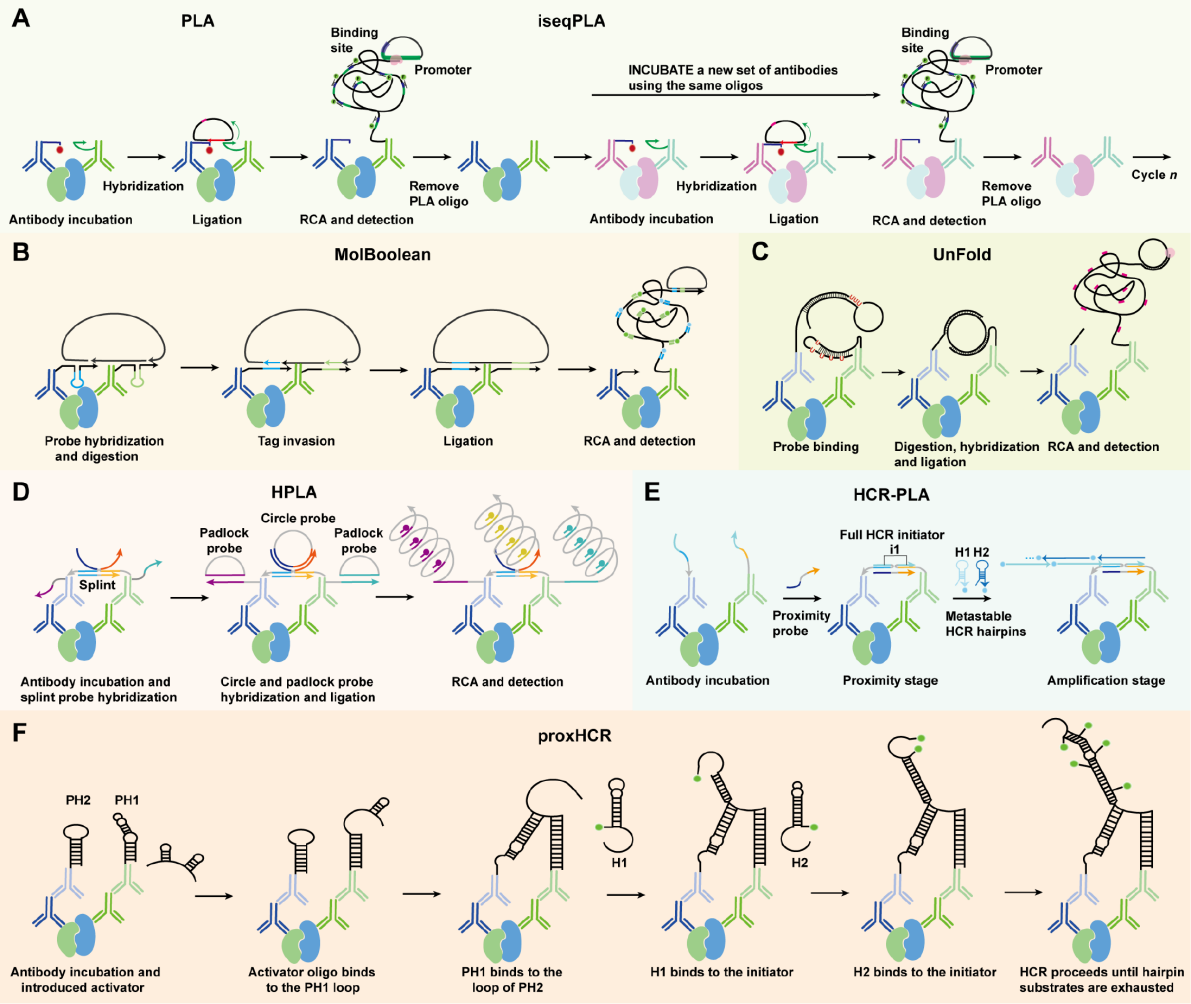
Figure 3 Principle of *in situ* PPI detection technology based on AOCs (A) PLA: two proximity probes bind to proteins in the same complex, forming a circular structure via DNA ligation, which undergoes RCA. The amplification products were visualized with fluorescent oligonucleotides. iseqPLA: PPIs are detected in cell cultures or tissue samples via primary antibodies bound to oligonucleotides. Probes are removed between cycles, and multiple PPIs are detected at the subcellular level. (B) MolBoolean: proximity probes A and B bind to their respective target proteins A and B and then hybridize to the circle. The circle is cleaved, invaded by a reporter tag, and enzymatically ligated. RCA generates long tandem products, which are detected by fluorescently labelled tag-specific oligonucleotides. (C) Unfold: probes are cleaved at U residues to form ligated DNA circles, which undergo RCA. The amplification products were visualized with fluorescent oligonucleotides. (D) HPLA: splint probes enhance DNA probe attachment to AOCs, promoting circularization, which undergoes RCA. The amplification products were visualized with fluorescent oligonucleotides. (E) HCR-PLA: a three-stage scheme where primary antibodies bind to proteins and proximity probes colocalize to trigger HCR amplification. The resulting fluorescent polymer is detected. (F) proxHCR: primary antibodies from different species bind their targets and are constrained by proximity probes. An activator oligonucleotide triggers HCR amplification, resulting in the production of fluorescently labelled products.

PPIs are pivotal in cellular signaling, gene regulation, and disease mechanisms, underscoring the need for precise detection methods in biological research and drug development [60]. Traditional approaches, such as co-immunoprecipitation and yeast two-hybrid assays [ 61, 62] , have been widely used in the study of PPIs but are limited by their inability to capture transient or weak interactions, high background noise, and lack of spatial resolution. These limitations have driven the development of advanced, spatially resolved, and highly specific technologies for *in situ* PPI detection, such as AOC-based proximity ligation assays (PLAs). PLA was first introduced as a revolutionary method for *in situ* PPI detection, leveraging antibodies conjugated to short DNA strands as probes with molecular proximity [63]. When two target proteins are within a distance of 30–40 nanometers, their conjugated DNA strands mediate the formation of a circular DNA template [ 64, 65] . This template is then amplified via RCA, generating a strong fluorescent signal that allows direct visualization of PPIs *in situ* ( Figure 3A). Despite its innovation, early PLA methods struggled with background noise from non-specific DNA hybridization and were limited by overlapping fluorescence signals, which restricted their utility in complex biological systems.

These shortcomings prompted the development of advanced PLA variants, each addressing specific technical limitations. First, intelligent sequential PLA (iseqPLA) expands this concept through multicycle detection with signal erasure between rounds, enabling high-throughput mapping of complex PPI networks with minimal crosstalk ( Figure 3A) [66]. To further enhance specificity and multiplexing capabilities, advanced PLA systems incorporate logic-based and cyclic detection strategies. Boolean logic-gated PLA, such as MolBoolean, ensures signal generation only when both proximity probes are present, effectively eliminating false positives ( Figure 3B) [67]. In addition, UnFold probe-based PLA keeps DNA strands in an inactive state until protein proximity triggers unfolding and amplification, further reducing background noise ( Figure 3C) [68]. Finally, hybridization-enhanced PLA (HPLA) employs a splint probe that hybridizes with the complementary DNA sequences of PLA probes bound to an interacting protein complex, forming a V-shaped overhang. This structure facilitates the circularization of two probes, thereby increasing the efficiency of PPI detection and enabling the simultaneous visualization of two proteins and their complexes ( Figure 3D) [69].

While these PLA variants primarily enhance detection specificity and multiplexing capabilities through probe and assay design, another major innovation lies in the signal amplification mechanism itself. HCR-PLA employs proximity probes designed using NUPACK for three orthogonal HCR amplifiers, enabling multiplexed imaging of three distinct protein complexes in A-431 human cells: α-tubulin:β-tubulin in the cytoskeleton, β-catenin:E-cadherin on the cell membrane, and SC35:SON in nuclear speckles ( Figure 3E) [70]. Furthermore, proxHCR builds upon traditional RCA-based PLA by combining PLA specificity with enzyme-free HCR amplification, significantly reducing assay costs and increasing the suitability for high-throughput screening of protein interactions ( Figure 3F) [71]. In addition to PLA, proximity barcoding assays and molecular pixelation use DNA barcoding to capture spatial protein co-localization without detecting direct interactions, offering scalable, sequencing-based alternatives to PLA for tissue-level protein mapping [ 72, 73] .

In summary, the rapid evolution of AOC-based proximity approaches has reshaped spatial proteomics by enabling sensitive, specific, and multiplexed PPI detection within native cellular contexts. Through the integration of programmable DNA barcodes, signal amplification, and computational logic, these methods unlock new opportunities in biomarker discovery, disease modelling, and precision medicine.

### AOC-mediated multimodal profiling

Simultaneous detection of proteins and nucleic acids is essential for decoding cellular complexity. AOC-based multimodal detection methods leverage programmable DNA barcodes to enable high-resolution, multiplexed imaging of proteins, RNA, DNA, and post-translational modifications *in situ* [ 74, 75] . By integrating advanced amplification and hybridization strategies, these techniques expand the molecular depth of spatial omics while preserving tissue architecture.

**Figure 4:**
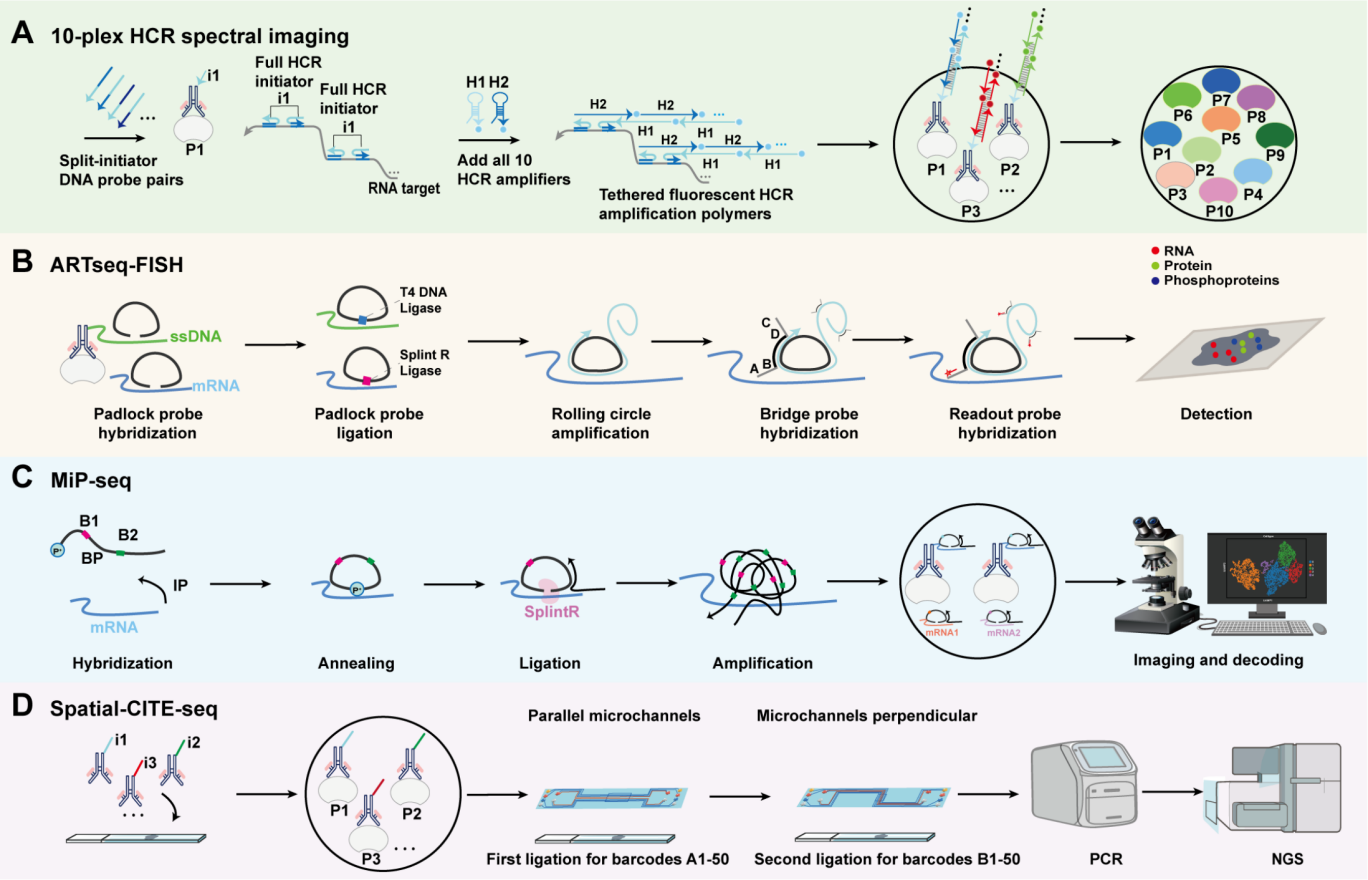
Figure 4 Principle of spatial multi-omics technology based on AOC (A) 10-plex HCR spectral imaging enables parallel in situ detection and amplification of proteins and RNAs. Antibody-conjugated DNA initiators bind to protein targets, while split-initiation DNA probes hybridize to RNA. The fluorophore-labelled HCR hairpins then self-assemble into amplification polymers, generating spatially resolved signals for all 10 targets simultaneously. (B) ARTseq-FISH enables the co-detection of proteins, phosphoproteins, and mRNAs in situ. AOCs target proteins, while padlock probes bind to corresponding mRNAs. RCA generates localized rolling-circle products, which are hybridized with fluorescent barcoded probes for multiplexed imaging. (C) MiP-seq involves padlock probe hybridization and in situ RCA for high-throughput spatial RNA profiling. Barcoded ligation-based sequencing enables signal decoding across multiple rounds, allowing comprehensive transcriptome mapping at subcellular resolution. (D) Spatial-CITE-seq utilizes a DNA-barcoded antibody panel to label 200–300 proteins in fixed tissue sections. Spatially defined barcoding via microfluidic channels generates a unique 2D positional code, which is reverse transcribed alongside endogenous mRNA. The resulting barcoded cDNA is sequenced, enabling integrated spatial proteomic analysis.

Initial RNA-protein co-detection was combined with RNA fluorescence *in situ* hybridization (FISH) with IF but was limited by low sensitivity and poor multiplexing. Alternative strategies such as single-molecule FISH (smFISH) [76] and RNAscope [77] have enhanced RNA detection resolution; however, their integration with protein detection remains technically challenging, and multiplexing is still constrained. To overcome this, combining AOCs with HCR enables non-enzymatic, highly specific signal amplification for the simultaneous detection of RNA and protein ( Figure 4A) [78]. In HCR-based AOC assays, DNA-conjugated antibodies and RNA probes trigger hybridization cascades, resulting in up to tenfold multiplexing per round. This approach is especially useful for mapping the tumor microenvironment and cellular states. Amplification-based single-molecule FISH combined with IF (asmFISH-IF) improves RNA detection efficiency while retaining spatial information [ 79, 80] .

Antibody-mRNA targeted sequential FISH (ARTseq-FISH) incorporates the detection of phosphorylated proteins into RNA-protein imaging [81], allowing trimodal visualization of transcripts, proteins, and signaling activities *in situ* for studying immune activation and oncogenic pathways ( Figure 4B). To further push the boundaries of multiplexing, multi-omics *in situ* pairwise sequencing (MiP-seq) enables the *in situ* co-detection of RNA, DNA, proteins, and neurotransmitters by employing sequential hybridization on AOC scaffolds ( Figure 4C) [74]. This is particularly powerful in complex tissues, such as the brain, where fine molecular compartmentalization is essential.

Digital spatial profiling (DSP) [82] enables high-throughput RNA-protein co-detection by employing antibodies or RNA probes conjugated to photocleavable oligonucleotide barcodes, which are spatially released upon UV illumination of selected tissue regions. These barcodes are then collected and quantified through optical imaging rather than sequencing, allowing non-destructive and multiplexed molecular profiling while preserving spatial context. In contrast, spatial co-indexing of transcriptomes and epitopes for multi-omics mapping by highly parallel sequencing (Spatial-CITE-seq) [83] relies on sequencing antibody-tagged or RNA-associated barcodes, providing large-scale multi-omics maps across tissues. While these approaches offer limited subcellular resolution, they are well-suited for profiling extensive tissue areas. Furthermore, they can be integrated with AOC-based imaging to bridge the gap between fine-resolution molecular localization and broad-scale tissue context ( Figure 4D). Collectively, these integrated approaches expand the spatial biology toolkit, enabling the scalable and high-resolution decoding of RNA-protein co-detection within complex tissue environments.

### Data analysis strategies for AOC-based spatial proteomics: from multiplex detection to interaction and multi-modal integration

**Figure 5:**
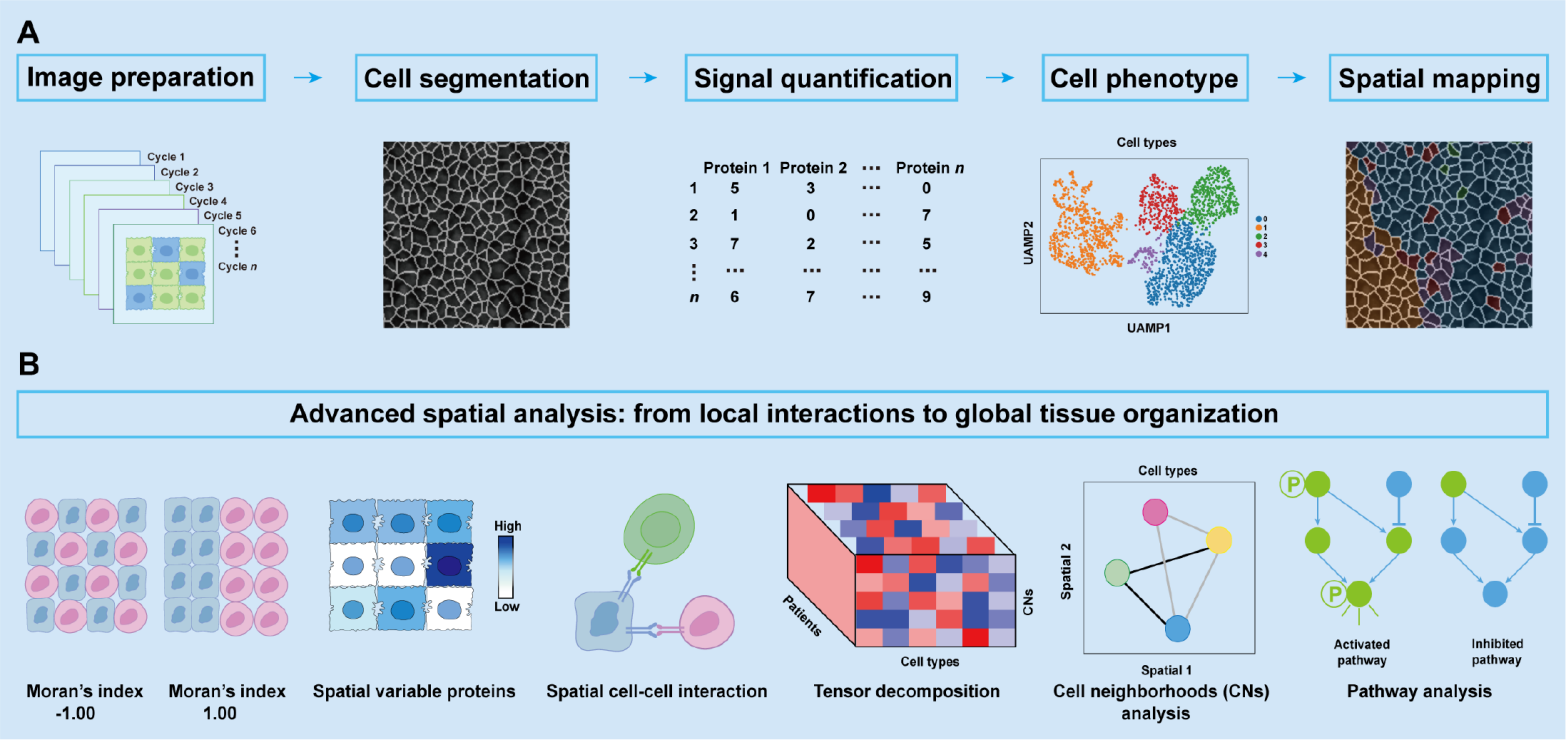
Figure 5 Computational workflow for AOC-based spatial proteomics (A) Preprocessing and basic cell-level analysis. The initial steps include image preprocessing, cell segmentation, signal recognition, and quantification. On the basis of these processed data, cells are phenotyped and mapped to reconstruct protein distributions across tissue architecture. (B) Advanced spatial analysis: from local interactions to global tissue organization. In addition to basic mapping, multiple approaches are used to extract high-dimensional spatial information. Neighborhood analysis and spatial interaction modelling quantify local cell-cell communication and co-occurrence patterns. Tensor decomposition reduces dimensionality and identifies latent spatial modules across markers or samples. Spatially variable protein analysis detects heterogeneously distributed proteins, offering insights into localized functional states and microenvironments. Pathway analysis integrates spatially resolved molecular profiles with biological networks to uncover functional programs underlying tissue organization. Spatial autocorrelation metrics (e.g., Moran’s I) assess the clustering or dispersion of molecular features, providing quantitative measures of tissue architecture.

AOC-based spatial proteomics platforms yield diverse signal types, including continuous fluorescence intensities, discrete molecular puncta, and hybrid barcoded readouts ( Supplementary Table S6). Each signal type requires tailored computational workflows that encompass image preprocessing, feature extraction, and spatial modelling. Despite variations in imaging modalities and amplification chemistries, core analytical steps are broadly conserved across platforms. These include image preprocessing, cell segmentation, signal quantification, and downstream spatial or single-cell analysis ( Figure 5)[ 55, 84] .

Image preprocessing typically involves steps such as drift correction, illumination normalization, and alignment across spectral channels or imaging cycles. Open-source tools, such as Fiji/ImageJ, BigWarp, BaSiC, and Elastix, are commonly used in this stage [ 85, 86] . Cyclic imaging systems, such as CODEX [25] and iseqPLA [66], require precise registration across imaging rounds to assign signals to the correct cells. This is often implemented through affine or non-rigid transformations using Python libraries such as scikit-image and OpenCV [ 87, 88] . Signal amplification platforms such as HCR-IHC [56], Immuno-SABER [57], and SABER-IMC [58] often rely on signal deconvolution and denoising to increase detection sensitivity. These steps are typically performed via custom Python or MATLAB pipelines alongside ImageJ/Fiji plugins [89]. Cell segmentation is a critical step and is usually performed using nuclear or membrane markers. Approaches may involve traditional rule-based workflows through tools such as CellProfiler and Ilastik or deep learning-based models such as Cellpose, StarDist, or DeepCell [ 90– 93] . The segmentation results can be interactively visualized and annotated using platforms such as Napari or QuPath. Once segmentation is completed, spatial expression matrices at the single-cell level are generated by extracting either signal intensities or spot counts, depending on the data modality [ 94, 95] .

Signal quantification strategies are contingent on the underlying signal type. Platforms such as CODEX and SeqStain [53] produce continuous intensity signals that require normalization across imaging rounds or batches. Common approaches include quantile normalization, Z-scoring, or batch correction using ComBat [ 96, 97] . In contrast, punctate-based methods such as the PLA [63], UnFold [68], HPLA [69], and MolBoolean [67] commonly utilize CellProfiler pipelines for spot detection and quantification, employing algorithms such as Laplacian-of-Gaussian filtering or object-based detection modules ( *e*. *g*., IdentifyPrimaryObjects). These workflows often involve per-cell quantification of puncta and calibration against negative controls or background fluorescence to increase specificity and reduce false positives [98].

Moreover, spatially resolved detection of PPIs introduces additional analytical complexity. Methods such as UnFold and MolBoolean aim to differentiate between single and dual proximity events, which requires subcellular-level co-localization analysis. The iseqPLA, which builds upon *in situ* PLA chemistry, accumulates signals over multiple imaging cycles and depends on precise spatial registration to ensure localization accuracy and signal integrity [99].

In addition to protein-only measurements, emerging AOC-based platforms support multi-modal integration with transcriptomic data. Multi-modal platforms, such as MiP-seq [74], ARTseq-FISH [81], and spatial-CITE-seq [83], as well as high-plex imaging systems such as 10-plex HCR [78], enable the simultaneous detection of proteins and transcripts. Analyzing these datasets requires the integration of spatially resolved protein data with transcriptomic reads. Alignment and joint analysis are typically performed using canonical correlation analysis, non-negative matrix factorization, or probabilistic models, such as totalVI [ 100– 102] . Widely used frameworks such as Seurat v4, Scanpy, and Squidpy provide toolkits for clustering, dimensionality reduction, spatial graph construction, and neighborhood analysis [ 103– 105] . For techniques such as 10-plex HCR, spectral unmixing is necessary to separate highly multiplexed signals. This is typically accomplished via linear unmixing, independent component analysis, or blind source separation techniques, which are implemented in ImageJ, MATLAB, or Python-based spectral analysis libraries [ 106, 107] .

In summary, the analysis of AOC-based spatial proteomics data integrates a wide range of computational approaches spanning image processing, single-cell data analysis, and spatial modelling. While general workflows are becoming more standardized, differences in signal characteristics and multiplexing chemistries across platforms still require the development of customized pipelines that effectively align imaging data with spatial and molecular context.

## Applications of AOC-based Spatial Proteomics

**Figure 6:**
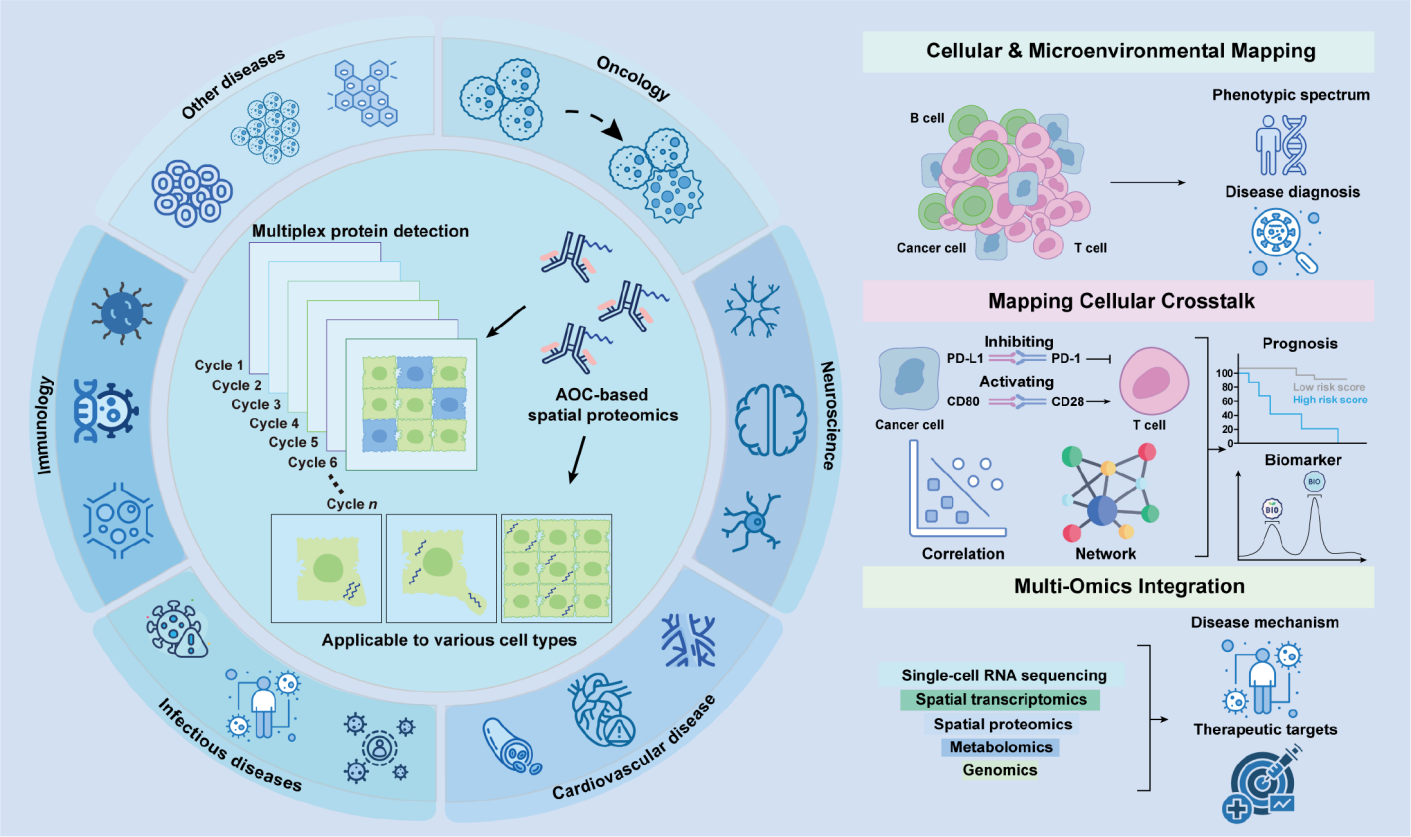
Figure 6 Applications of AOC-based spatial proteomics The figure illustrates the applications of AOC-based spatial proteomics in oncology, neuroscience, infectious disease, cardiovascular pathology, immunology, and other conditions, highlighting three analytical dimensions: cellular and microenvironmental mapping, mapping of cellular crosstalk, and multiomics integration. Cellular and microenvironmental mapping enables disease typing and diagnosis by charting the spatial organization of cells and their microenvironments; mapping of cellular crosstalk, combined with correlation analysis and network modelling, supports prognosis assessment and biomarker discovery; and multi-omics integration with AOC-based spatial proteomics facilitates the exploration of disease mechanisms and identification of potential therapeutic targets.

AOC-based spatial proteomics facilitates not only the identification of spatially distinct cell states but also the detection of PPIs within intact tissue architecture. When integrated with transcriptomic and other omics data, AOC-based approaches provide a multidimensional view of pathological processes, with broad applications in oncology, neuroscience, infectious disease, cardiovascular pathology, autoimmunity, and other areas ( Figure 6).

### Cellular and microenvironmental mapping with AOC-based spatial proteomic technology

AOC-based spatial proteomics has enabled the precise delineation of cellular phenotypes and spatial organization across multiple tissue types under healthy and diseased conditions. By capturing *in situ* protein expression at single-cell resolution, this approach facilitates the identification of tissue microstructures and pathophysiological niches [108]. For example, in hepatocellular carcinoma, a 36-AOC panel profiled 401 human samples, identifying co-localization of vimentin ^high^ macrophages and Tregs that correlate with immunotherapy response and disease progression [109].

In Alzheimer’s disease (AD), CODEX imaging revealed region-specific distributions of microglia and their association with tau pathology in affected brain areas, shedding light on the spatial dynamics of neuroinflammation [110]. For tuberculosis, AOC-based spatial proteomics was used to examine granulomas, where lesion-resident macrophages and CD20 ^+^ B cells were found to contribute to protective immune architecture [111]. In abdominal aortic aneurysm (AAA), spatial proteomics provides a cellular atlas of immune and stromal cell populations and reveals C-reactive protein (CRP)-associated shifts in immune cell localization related to vascular remodeling [112].

In diabetic kidney disease, this technology identified SPP1 as a proximal-distal tubular signaling mediator implicated in early tubular injury [113]. Similarly, in pemphigus, CODEX revealed organized autoreactive immune structures within tertiary lymphoid sites [114]. During lung organogenesis, spatial proteomics revealed epithelial and immune cell lineage trajectories, enhancing our understanding of developmental immune maturation [115].

### Mapping cellular crosstalk using AOC-based spatial proteomics

AOC-based spatial proteomics extends beyond protein expression profiling to enable *in situ* detection of PPIs with molecular resolution in native tissue contexts, revealing previously inaccessible interaction networks while preserving spatial organization. For example, in AD, PLA revealed site-specific ubiquitin-modified phosphorylated tau aggregates, offering mechanistic insights into disease pathology [116]. In early-stage non-small cell lung cancer (NSCLC), Unfold detected functional PD-1/PD-L1 interactions that correlated more closely with the immune checkpoint inhibitor response and patient survival than PD-L1 expression alone. This functional assay is compatible with routine clinical samples and supports semiquantitative analysis, underscoring its potential to inform immunotherapy decisions [117].

In cardiovascular applications, ProteinSeq extends spatial proteomics to liquid biopsy by enabling high-sensitivity plasma protein detection. A clinical study employing ProteinSeq identified three novel biomarkers for cardiovascular risk stratification (P-selectin, Cystatin-Band, and Kallikrein-6), demonstrating its translational relevance [118]. The combination of CODEX with *in situ* PLA has captured microenvironmental shifts induced by CRP accumulation in AAA, highlighting the inflammatory networks driving vascular remodeling [ 112, 119, 120] .

The integration of AOC-based multiplexing with spatially resolved interaction detection not only enhances the characterization of cell states but also facilitates the identification of novel therapeutic targets. From tumor immune evasion mechanisms to neurodegenerative signaling cascades and infectious immune synapses, these applications underscore the versatility of spatial PLA and proximity assays in decoding complex biological communication.

### Applications of AOCs in multi-omics analyses

The fusion of AOC-based spatial proteomics with other omics platforms, such as single-cell RNA sequencing (scRNA-seq), spatial transcriptomics (ST), and epigenomic profiling, has ushered in a new era of systems tissue biology. In NSCLC, the integration of CODEX, scRNA-seq (~900,000 cells), and STs from 8 patients enabled functional annotation of spatial immune heterogeneity and tumor-associated macrophages-mediated metabolic adaptation [121]. In breast cancer, cross-modality integration captures subtype-specific chromatin landscapes and localized immune infiltration, linking gene accessibility with proteomic spatial signatures [122].

In infectious diseases, such integrative approaches have improved the understanding of host-pathogen interactions. Tuberculosis studies combining CODEX and scRNA-seq revealed dense intercellular signaling between IL4I1 ^+^ myeloid cells and Tregs in granulomas [123]. Moreover, in coronavirus disease 2019, CODEX disrupted germinal center reactions and impaired Bcl-6 ^+^ T follicular helper responses, explaining poor antibody durability [124]. These multimodal findings provide mechanistic explanations for immune evasion and therapeutic failure. In cardiovascular disease, spatial proteomic data combined with histopathology and transcriptomic atlases have mapped inflammation across vascular territories, providing granular insight into atheroprogression and AAA evolution [ 125, 126] .

Furthermore, CODEX-based studies now support retrospective analyses using archived FFPE samples, enabling long-term cohort studies across disease types. In developmental biology, the integration of spatial proteomics with single-cell sequencing has clarified the spatiotemporal differentiation trajectories in lung and bone marrow development [ 115, 127] . As machine learning (ML) and spatial analytics tools mature, such integrative frameworks will enable the reconstruction of dynamic tissue ecosystems and predictive modelling of disease progression and therapy response. Taken together, the integration of AOC-driven spatial proteomics with multi-omics approaches provides a powerful lens for resolving the molecular architecture of human disease.

## Current Limitations and Prospects of AOC-based Spatial Proteomics

Despite remarkable progress, several challenges continue to limit the accuracy, reproducibility, and scalability of AOC-based spatial proteomics. Among these factors, antibody-related factors remain critical. Specificity, affinity, and compatibility with DNA conjugation chemistry directly affect signal quality and consistency [128]. As discussed in the antibody selection section, careful validation and preparation are essential to ensure robustness across experimental contexts.

In addition to antibody selection, accurate signal acquisition remains a key technical challenge in AOC-based spatial proteomics. Multiplexed assays must resolve highly similar protein isoforms and detect low-abundance targets, which are often hindered by steric crowding [129]. These difficulties are further amplified by issues in tissue processing, including fixation, antigen retrieval, and sectioning, which impact antigen stability and accessibility [130]. Imaging artefacts such as autofluorescence and uneven staining can also obscure true biological signals. Addressing these issues requires improved barcode designs, refined tissue protocols, and advances in microscopy and signal deconvolution to ensure spatial fidelity and analytical depth [ 125, 131] .

The computational analysis of AOC-based data presents additional obstacles. These workflows generate large-scale, high-dimensional datasets that demand sophisticated algorithms for integration, normalization, and interpretation [132]. Challenges include batch correction, signal normalization, and the integration of proteomic data with other omics layers. Recent developments in ML and artificial intelligence (AI) are enabling more accurate cell segmentation, spatial pattern recognition, and network inference, expanding the interpretive power of spatial proteomics [133].

Innovations in antibody engineering, DNA barcoding, microfluidic conjugation, and computational pipelines are rapidly increasing the resolution, sensitivity, and throughput of AOC-based spatial proteomics. Integrative strategies that combine spatial proteomics with transcriptomics and epigenomics are driving the development of next-generation multi-omics platforms for precision medicine [134]. In parallel, efforts to reduce technical barriers such as cost, scalability, and accessibility are making these technologies more widely adoptable. Open-source tools and streamlined protocols are expanding their reach to diverse research settings, including those with limited resources [135].

### Integration of AOC-based spatial proteomics with AI

The integration of AI with AOC-based spatial proteomics is transforming the analysis of complex biological systems. By enhancing data acquisition, processing, and interpretation, these technologies facilitate the robust and scalable extraction of insights from high-dimensional, spatially resolved proteomic datasets.

Spatial proteomics generates large, information-rich images and spatial maps that require advanced algorithms to extract meaningful biological insights. Several ML-based tools have emerged to streamline this process. One notable breakthrough is ML for the analysis of proteomics in spatial biology (MAPS), which employs ML algorithms to classify cell types, protein networks, and tissue architectures with near-pathology-level accuracy. Compared with traditional methods, which are validated on both internal and publicly available CODEX datasets, MAPS has demonstrated superior speed and precision in terms of cell type annotation and spatial protein distribution analysis [136]. Additionally, the spatial graph Fourier transform (SpaGFT) [137] and single-cell graph convolutional operator (scGCO) [138] are sophisticated computational techniques designed to increase the spatial resolution and precision of protein localization studies. These approaches enable more precise mapping of PPIs and the tumor microenvironment, providing a more refined view of their cellular organization and tissue structure.

Deep learning architectures have also significantly improved imaging resolution and data interpretation in spatial proteomics. DeepSearch optimizes database search processes in tandem MS, increasing protein identification sensitivity and resolution [139]. Moreover, spatial proteomics image deconvolution by super-resolution (SpiDe-Sr) integrates self-supervised denoising and blind super-resolution networks, effectively addressing imaging noise and resolution constraints in IMCs. SpiDe-Sr achieves a fourfold enhancement in spatial resolution, improving cell segmentation and clustering accuracy, which is critical for analyzing intricate tissue structures [140]. These innovations are crucial for mitigating challenges such as imaging noise and artefacts, thereby ensuring the robustness and reproducibility of spatial proteomic analyses.

The integration of spatial proteomics with multi-omics datasets is another frontier where AI is playing a pivotal role. Multi-modal spatial omics (MISO) [141] and parallel-flow projection and transfer learning across omics data (PLATO) [133] are two powerful frameworks that integrate proteomics with transcriptomics, genomics, and epigenomics, enabling a comprehensive multi-omics landscape of disease states. MISO focuses primarily on joint embedding and co-visualization of heterogeneous spatial omics modalities, thereby facilitating the discovery of coordinated molecular programs across layers of regulation. In contrast, PLATO employs transfer learning-based projections to align proteomic profiles with other omics datasets, enhancing cross-modal prediction and enabling the detection of spatially distinct tumor subtypes and dysregulated proteins in cancer. These AI-powered tools provide a systems-level understanding of tissue microenvironments, facilitating the discovery of biomarkers and the identification of therapeutic targets.

As computational strategies continue to advance alongside next-generation imaging and multiplexed antibody panels, the integration of AI with AOC-based spatial proteomics is poised to drive groundbreaking discoveries in developmental biology, precision oncology, and characterization of the immune microenvironment. The next step in this evolution will be closing the loop between AI-driven predictions and experimental validation, creating iterative workflows in which AI-generated hypotheses, such as PLATO’s dynamic modelling of tumor heterogeneity or SpiDe-Sr’s super-resolution reconstructions that improve cell segmentation and tissue architecture mapping, direct experimental perturbations in spatial proteomics. Such bidirectional approaches will not only increase algorithmic accuracy but also accelerate the translation of spatial proteomic insights into therapeutic innovations, ultimately bridging the gap between computational modelling and real-world clinical applications.

### Potential applications of AOC-based spatial proteomics

AOC-based spatial proteomics holds immense potential in drug development and precision medicine, enabling a more comprehensive understanding of therapeutic effects at the molecular and cellular levels [142]. Integrating AOC-based spatial proteomics with pharmacological research allows for *in situ* protein-level analyses of drug responses, offering an innovative approach to identifying novel drug targets, predicting drug efficacy, and elucidating complex signaling pathways involved in disease progression [143]. This is particularly valuable for studying highly heterogeneous diseases, such as cancer, autoimmune disorders, and neurodegenerative diseases, where spatial proteomic insights can guide patient stratification, drug resistance mechanisms, and personalized treatment strategies.

While spatial proteomics remains underutilized in natural medicine research, its ability to generate postdrug proteomic landscapes offers a novel framework for evaluating multi-component therapeutics, such as those found in traditional Chinese medicine. By profiling *in situ* protein expression changes, AOC-based spatial proteomics can help uncover synergistic effects, identify off-target interactions, and elucidate novel pharmacological mechanisms, thereby optimizing drug formulations and improving treatment outcomes [144]. This approach is particularly relevant for herbal and combination therapies, where the molecular mechanisms often remain poorly understood owing to the complexity of the active ingredients.

As preclinical models have evolved to better mimic human physiology, AOC-based spatial proteomics provides spatially resolved insights into tissue architecture, cellular interactions, and dynamic molecular changes within human organs [145]. This is especially critical for advancing organoids, patient-derived xenografts, and *ex vivo* tissue models, which serve as key bridges between *in vitro* studies and clinical applications [146]. However, several technical challenges remain, including limited imaging resolution and inconsistencies in sample preparation, which continue to restrict the application of AOC-based spatial proteomics in complex three-dimensional (3D) structures. Overcoming these limitations will require improvements in high-resolution imaging, AI-assisted data analysis, spatial multi-omics integration, and computational modelling, all of which are essential for refining tissue-based pharmacological research and accelerating the development of therapeutics.

Future innovations in spatiotemporal proteomics, 3D tissue mapping, and large-scale human atlases will continue to extend the capabilities of AOC-based spatial proteomics, offering molecular-resolution insights into human health and disease. These advances will support its integration into precision medicine by enabling more dynamic and spatially resolved analyses of disease progression and therapeutic response. Realizing this potential will require continued progress in methodology, data integration, and cross-disciplinary innovation.

## Conclusions and Perspectives

AOCs have emerged as a transformative tool in spatial proteomics, offering highly specific, multiplexed protein detection and spatial mapping within complex tissue microenvironments. By integrating antibody-based recognition with DNA barcoding and amplification, AOCs enable sensitive, single-molecule resolution imaging and are compatible with sequencing-based readouts and proximity assays. This molecular programmability also facilitates cross-modal analyses, allowing simultaneous detection of proteins alongside RNA and DNA to comprehensively profile cellular signaling networks.

AOC-based spatial proteomics has found broad applications in oncology, neuroscience, infectious disease, and cardiovascular research, revealing key biomarkers, therapeutic targets, and spatially resolved interactions within tumor microenvironments, inflamed neural circuits, infected tissues, and remodeling vasculature. Integration with tools such as PLA, CODEX, Immuno-SABER, and spatial transcriptomics has further expanded their utility for probing PPIs, post-translational modifications, and immune contextures at single-cell and subcellular resolutions.

Despite these advances, technical challenges persist, including variability in antibody performance, suboptimal conjugation and amplification efficiency, limited resolution in densely packed tissues, and the complexity of analyzing high-dimensional data. Addressing these issues will require validated monoclonal antibody libraries, standardized conjugation protocols, and next-generation barcoding strategies to ensure reproducibility and analytical robustness. On the computational side, AI and ML are increasingly being leveraged for image analysis, denoising, and spatial feature extraction, enabling automated and scalable proteomic workflows.

The continued evolution of AOC-based spatial proteomics will benefit from deeper integration with other omics studies, including genomics, transcriptomics, epigenomics, and metabolomics. Innovations in 3D spatial proteomics, spatiotemporal protein mapping, and real-time tissue profiling will further expand their impact on precision medicine, drug discovery, and immuno-oncology, ultimately accelerating the clinical translation of spatial proteomics.

## Supporting information

25626Supplementary_Tables

## Supplementary Data

Supplementary data are available at *Acta Biochimica et Biophysica Sinica* online.
