# Supplementary material for: Antibody-oligonucleotide conjugates for spatial proteomics: principles, applications, and challenges: Antibody-oligonucleotide conjugates for spatial proteomics
Source: Acta Biochim Biophys Sin (Shanghai). 2025 Nov 27;58(5):947–62. doi: 10.3724/abbs.2025212 (PMC13191458; doi:10.3724/abbs.2025212)
Supplement: 25626Supplementary_Tables [file 25626Supplementary_Tables.docx]

| **Supplementary Table S1. Comparison of antibody formats for AOC applications** | | | | | | |
| --- | --- | --- | --- | --- | --- | --- |
| Antibody format | Molecular weight | Specificity and affinity | Conjugation compatibility | Commercial availability | Structural advantages | Potential limitations |
| IgG (Monoclonal) | ~150 kDa | High specificity and affinity | Stable and efficient | Widely available | Well-characterized, structurally stable | Large size may limit epitope accessibility |
| IgG (Polyclonal) | ~150 kDa | Broad epitope recognition | Variable batch-to-batch | Readily available | Enhanced signal via multi-epitope binding | Lower reproducibility, higher background |
| IgM | ~900 kDa | Very high avidity | Low conjugation efficiency | Moderately available | Multivalent binding | Large and complex structure, difficult to modify |
| Nanobody | ~15 kDa | High specificity | Highly compatible with conjugation | Increasingly available | Small, stable, excellent tissue penetration | May have lower intrinsic affinity |
| VNAR (Shark-derived) | ~12 kDa | Able to bind cryptic epitopes | Extremely stable | Limited availability | Smallest known antigen-binding unit | Rare, requires custom development |
| Fab Fragment | ~50 kDa | High specificity | Suitable for site-specific methods | Commercially available | Smaller than IgG, easier to conjugate | Lacks Fc domain, shorter serum half-life |
| scFv | ~25-30 kDa | Tunable affinity through engineering | Suitable for site-specific methods | Expressible in various systems | Modular, customizable | May have lower stability or require optimized folding |

| **Supplementary Table S2. Comparison of lysine vs. cysteine conjugation** | | |
| --- | --- | --- |
| Feature | Lysine conjugation | Cysteine conjugation |
| Mechanism | NHS ester-amino reaction | Maleimide-thiol reaction |
| Conjugation Sites | Multiple lysines (~40 per IgG) | Limited cysteines (~12 per IgG) |
| Labeling Uniformity | Heterogeneous | More site-specific |
| Antibody Affinity Impact | Potential steric hindrance | Minimal interference |
| Stability | Moderate | High (thioether bonds) |
| Technical Complexity | Simple | More complex (requires reduction) |
| Efficiency | High initial rate but prone to over-labeling | High yield with controlled site targeting |
| Reproducibility | Moderate, affected by lysine accessibility | High, due to defined reactive sites |
| Compatibility | Compatible with most buffers except amine-containing | Sensitive to reducing agents, otherwise broadly compatible |

| **Supplementary Table S3. Applicability of different click chemistry methods** | | |
| --- | --- | --- |
| Reaction | Key features | Applications |
| SPAAC | Copper-free, biocompatible, widely used | General AOC synthesis |
| IEDDA | Ultra-fast kinetics, no protective groups needed | Live-cell labeling |
| SPANC | High stability, low reagent concentrations | Intracellular labeling |
| CB-AAC | Mechanically interlocked molecules | Specialized structural studies |
| CCS | Enhanced reaction efficiency via hydrogen bonding | Broad substrate scope |

| **Supplementary Table S4. DNA-based amplification methods** | | | | | |
| --- | --- | --- | --- | --- | --- |
| Method | Suitable for *in situ* | Temperature | Mechanism | Strengths | Limitations |
| PCR | No | High (up to 95 °C) | Enzymatic cycling | High sensitivity and specificity | Requires thermal cycling; not compatible with tissue morphology |
| RCA | Yes | Mild (around 37 °C) | Isothermal enzymatic extension | High spatial localization; suitable for single-molecule detection | Dependent on circular template design and enzyme performance |
| HCR | Yes | Room temperature to 37 °C | Enzyme-free hybridization cascade | Low background; spatially confined; simple design | Moderate amplification efficiency; requires careful probe design |
| LAMP | No | Elevated (60–65 °C) | Isothermal strand displacement | Rapid and efficient amplification | High temperature; complex products; limited spatial resolution |

| **Supplementary Table S5. Summary of AOC-based spatial proteomics technologies** | | | | | | | | | |
| --- | --- | --- | --- | --- | --- | --- | --- | --- | --- |
| Method | Year | Antibody staining | Probe detection | Sample | Multiple testing capability | Antibody cross-linking sites | Crosslinking agent | DNA modification sites | Key reference |
| Multiplex protein detection | | | | | | | | | |
| DEI | 2017 | All-in-one | Cyclic | Cells, FF | >10 | Lysine residues and NHS ester | SM(PEG)2 (Thermo Fisher Scientific) | Maleimide and thiol-modified DNA | DOI: 10.1021/acs.nanolett.7b02716 |
| Immuno-SABER | 2019 | All-in-one | Cyclic + Amplification | Cells, FF, FFPE | >10 | Lysine residues and NHS ester | SM(PEG)2 (Thermo Fisher Scientific) | Maleimide and thiol-modified DNA | DOI: 10.1038/s41587-019-0207-y |
| CODEX | 2021 | All-in-one | Cyclic | Cells, FFPE | >60 | Cysteine and thiol | TCEP (Thermo Fisher Scientific) | Maleimide-modified DNA | DOI: 10.1038/s41596-021-00556-8 |
| SeqStain | 2021 | Cyclic | Cyclic | Cells, FF | >25 | Lysine residues and NHS ester | DBCO-Sulfo-NHS Ester ((Click Chemistry Tools) | DBCO and azide-modified DNA | DOI: 10.1016/j.crmeth.2021.100006 |
| HCR-IHC | 2021 | All-in-one | Cyclic + Amplification | Cells, FFPE | 10 | Lysine residues and S-HyNic | Antibody–Oligonucleotide All-in-One™ Conjugation Kit (VectorLabs) | S-4FB + amine-modified DNA | DOI: 10.1242/dev.199847 |
| CAD-HCR | 2022 | All-in-one | Cyclic + Amplification | Cells | 10 | Lysine residues and NHS ester | Sulfo-SMCC (Thermo Fisher Scientific) | Maleimide and thiol-modified DNA | DOI: 10.1126/sciadv.abk0133 |
| SABER-IMC | 2023 | All-in-one | Cyclic + Amplification | Cells, FFPE | >38 | Lysine residues and NHS ester | SM(PEG)2 (Thermo Fisher Scientific) | Maleimide and thiol-modified DNA | DOI: 10.1038/s41592-023-01976-y |
| Protein-protein interactions | | | | | | | | | |
| PLA | 2006 | All-in-one | RCA | Cells, FF | 1 | Lysine residues and NHS ester | Sulfo-SMCC (Thermo Fisher Scientific) | Maleimide and thiol-modified DNA | DOI: 10.1038/nmeth947 |
| proxHCR | 2015 | All-in-one | RCA | Cells, FF, FFPE | 1 | Lysine residues and NHS ester | SANH (VWR) | Amino and aldehyde-modified DNA | DOI: 10.1038/ncomms8294 |
| UnFold | 2018 | All-in-one | RCA | Cells, FFPE | 1 | Lysine residues and NHS ester | S-HyNic Linker (VectorLabs) | 6-Hydrazinonicotinic acid and aldehyde-modified DNA | DOI: 10.1038/s41598-018-23582-1 |
| MolBoolean | 2022 | All-in-one | RCA | Cells, FFPE | 2 | Lysine residues and NHS ester | S-HyNic Linker (VectorLabs) | 6-Hydrazinonicotinic acid and aldehyde-modified DNA | DOI: 10.1038/s41467-022-32395-w |
| HCR-PLA | 2023 | All-in-one | HCR | Cells, FFPE | 3 | Lysine residues and S-HyNic | Antibody–Oligonucleotide All-in-One™ Conjugation Kit (VectorLabs) | S-4FB + amine-modified DNA | DOI: 10.1021/acschembio.3c00431 |
| iseqPLA | 2024 | Cyclic | Cyclic + RCA | Cells, FF, FFPE | >47 | NA | Duolink and Navinci | NA | DOI: 10.1038/s41551-024-01271-x |
| HPLA | 2024 | All-in-one | RCA | Cells, FF, FFPE | 3 | Lysine residues and NHS ester | DBCO-PEG4-NHS (Sigma-Aldrich) | DBCO and azide-modified DNA | DOI: 10.1021/acs.analchem.4c04229 |
| Other approaches | | | | | | | | | |
| Spatial-CITE-seq | 2022 | All-in-one | PCR | FF, FFPE | >300 | Streptavidin and biotin | LYNX Rapid Streptavidin Antibody Conjugation Kit (Bio-Rad) and EZ Biotin S-S NHS (Thermo Fisher Scientific) | NHS ester and amine-modified DNA | DOI: 10.1101/2022.04.01.486788 |
| MiP-seq | 2024 | All-in-one | Cyclic + RCA | Cells, FF, FFPE | >30 | Lysine residues and NHS ester | NHS-PEG4-Azide (Thermo Fisher Scientific) | Azide and DBCO-modified DNA | DOI: 10.1038/s41551-024-01205-7 |
| ARTseq-FISH | 2024 | All-in-one | Cyclic + RCA | Cells, FFPE | >67 | Lysine residues and NHS ester | DBCO-S-S-NHS (Sigma-Aldrich) | DBCO and azide-modified DNA | DOI: 10.1038/s41467-024-48107-5 |
| 10-plex HCR spectral imaging | 2024 | All-in-one | HCR | FF | 10 | Lysine residues and S-HyNic | Antibody–Oligonucleotide All-in-One™ Conjugation Kit (VectorLabs) | S-4FB + amine-modified DNA | DOI: 10.1242/dev.202307 |

| **Supplementary Table S6. Comparison of data analysis features across AOC-based spatial proteomics methods** | | | | |
| --- | --- | --- | --- | --- |
| Method | Signal type | Multi-modal integration | Segmentation strategy | Common tools |
| DEI | Continuous | No | No explanation | Fiji, MATLAB |
| Immuno-SABER | Continuous (amplified) | No | Nucleus/membrane, watershed | Python, Cellprofiler, MATLAB |
| CODEX | Continuous | No | Nucleus/membrane, watershed | ImageJ, Microvolution, VorteX, CellEngine, Python, R |
| SeqStain | Continuous | No | Nucleus/membrane, StarDist | Cellprofiler, ImageJ, HALO |
| HCR-IHC | Continuous (amplified) | No | No explanation | Details are provided in section S2.6 of the original article |
| CAD-HCR | Continuous (amplified) | No | No explanation | ImageJ |
| SABER-IMC | Continuous (metal signal) | No | Nucleus/cytoplasmic, Ilastik | Cellprofiler, R |
| PLA | Discrete | No | No explanation | MATLAB |
| proxHCR | Continuous (amplified) | No | No explanation | Cellprofiler |
| HCR-PLA | Continuous (amplified) | No | No explanation | Details are provided in section S1.8 of the original article |
| UnFold | Discrete | No | No explanation | Cellprofiler |
| iseqPLA | Discrete | No | Nucleus/cytoplasmic, cellpose, deepcell | Python |
| HPLA | Discrete | No | No explanation | Cellprofiler |
| MolBoolean | Discrete | No | Nucleus/membrane | Cellprofiler |
| Spatial-CITE-seq | Continuous (seq counts) | Yes | No explanation | Python, R |
| MiP-seq | Continuous (mixed) | Yes | Nucleus/membrane, cellpose | Python, R |
| ARTseq-FISH | Continuous (mixed) | Yes | Al Based lterative Segmentation | Python |
| 10-plex HCR spectral imaging | Continuous (mixed) | Optional | No explanation | Details are provided in section S1.4 of the original article |
| **Note:** signal type: Continuous = fluorescence intensity; Discrete = spot counts or barcoded molecules | | | | |
